# Supplementary material for: Synthesis and Investigation of Novel CHCA-Derived Matrices for Matrix-Assisted Laser Desorption/Ionization Mass Spectrometric Analysis of Lipids
Source: Molecules. 2022 Apr 15;27(8):2565. doi: 10.3390/molecules27082565 (PMC9028824; doi:10.3390/molecules27082565)
Supplement: Supplementary file 1 [file molecules-27-02565-s001.zip › molecules-1644565-supplementary.pdf]

Synthesis and investigation of novel CHCA-derived matrices for matrix-assisted laser desorption/ionization mass spectrometric analysis of lipids

Antonio Monopoli[1]*, Giovanni Ventura[1], Andrea Aloia[1], Fulvio Ciriaco[1], Angelo Nacci[1,2], Tommaso R.I. Cataldi[1,3], Cosima D. Calvano[1,3]*

[1]Dipartimento di Chimica, Università degli Studi di Bari Aldo Moro, Via Orabona, 4 - 70126 Bari (Italy)

[2]CNR—Istituto di Chimica dei Composti Organometallici (ICCOM), Bari Section, Università degli Studi di Bari Aldo Moro, Via Orabona, 4 - 70126 Bari (Italy)

[3]Centro Interdipartimentale di Ricerca S.M.A.R.T., Università degli Studi di Bari Aldo Moro, Via Orabona, 4 - 70126 Bari (Italy)

Contents:

Figure S1. Positive MALDI-ToF MS spectra of the TAG mixture composed of trilaurin (LLL, 638.55 Da), trimyristin (MMM, 722.64 Da), tripalmitin (PPP, 806.74 Da), and tristearin (SSS, 890.83 Da) mixed with examined compounds **1** (A), **2** (B), **6** (C) and **7** (D).

Figure S2. The performance dependence in terms of S/N ratio on the proton affinities (red circles), extinction coefficients (blue circles), and logP (green circles) of each matrix applied to phosphatidylcholine (A), phosphatidylethanolamine (B), and triacylglycerol (C). Matrix **11** refers to CHCA while **12** corresponds to ClCCA.

Figures S3-S12. NMR Spectra

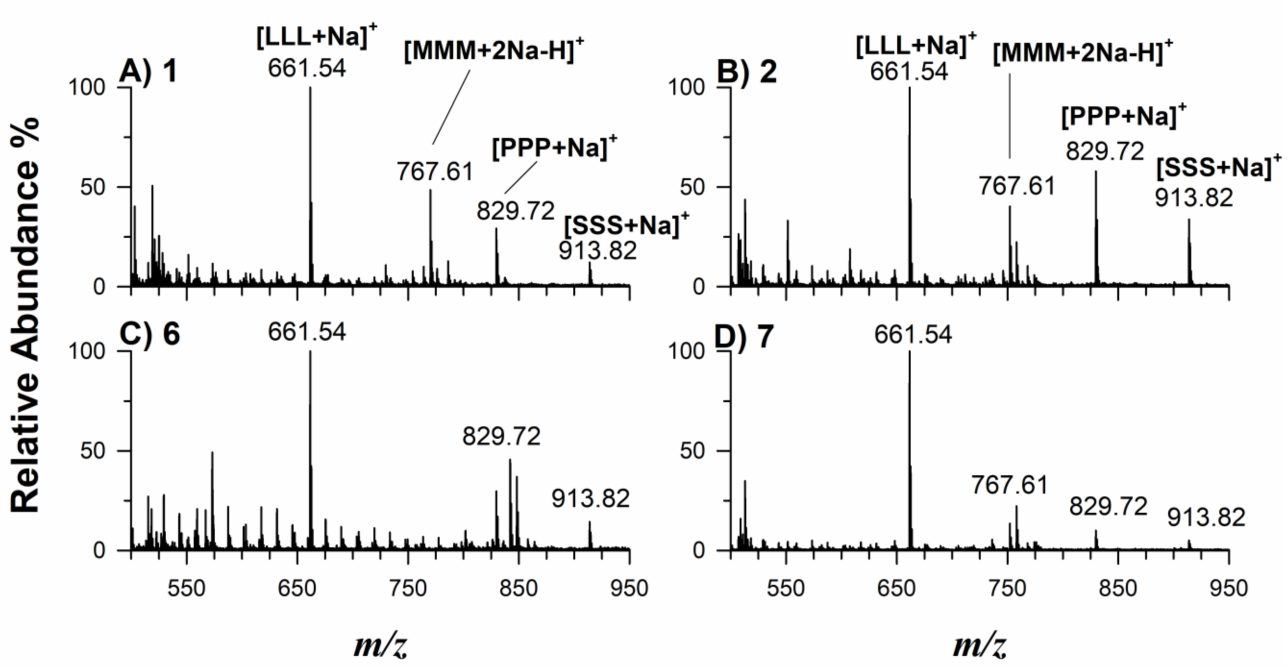

Figure S1. Positive MALDI-ToF MS spectra of the TAG mixture composed of trilaurin (LLL, 638.55 Da), trimyristin (MMM, 722.64 Da), tripalmitin (PPP, 806.74 Da), and tristearin (SSS, 890.83 Da) mixed with examined compounds **1** (**A**), **2** (**B**), **6** (**C**) and **7** (**D**).

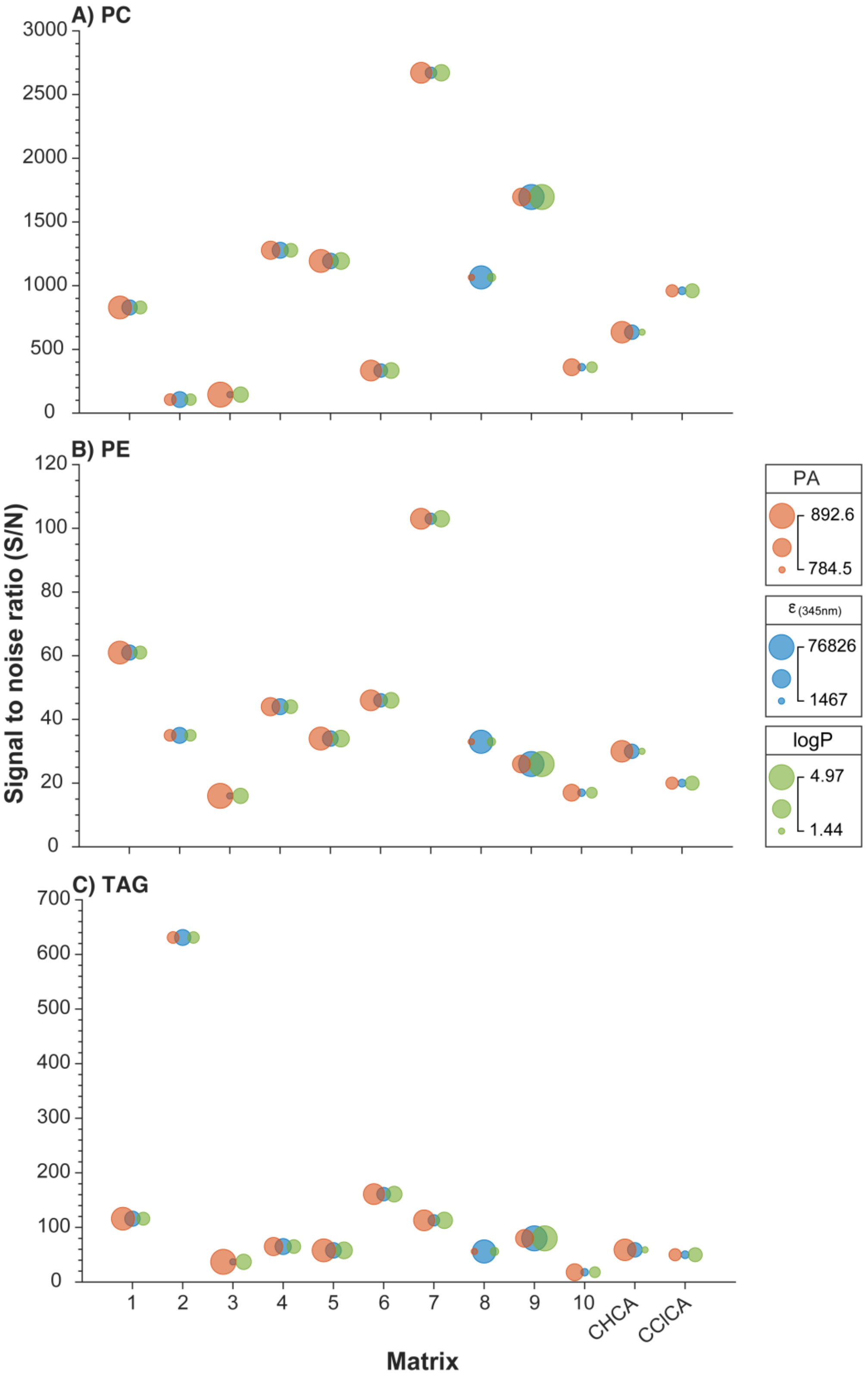

Figure S2. The performance dependence in terms of S/N ratio on the proton affinities (red circles), extinction coefficients (blue circles), and logP (green circles) of each matrix applied to phosphatidylcholine (**A**), phosphatidylethanolamine (**B**), and triacylglycerol (**C**). Matrix **11** refers to CHCA while **12** corresponds to ClCCA.

<u>*(2E,4E)-2-cyano-5-(4-methoxyphenyl)penta-2,4-dienoic acid (**1**)*</u>. This acid was obtained as a pale-yellow solid (m.p. 246-248°C) in a 82% of yield. ^{1}H-NMR (400 MHz, DMSO-D6), δ: 3.82 (s, 3H, methoxy), 7.02 (d, 2 H, J= 8.80 Hz, aromatic protons), 7.07 (dd, 1 H, J= 15.16 and 11.74 Hz, vinyl proton), 7.59 (d, 1 H, J= 15.16 Hz, vinyl proton), 7.67 (d, 2 H, J= 8.80 Hz, aromatic protons), 8.05 (d, 1 H, J= 11.74 Hz, vinyl proton); ^{13}C-NMR (DMSO-D6) δ: 163.37, 161.63, 155.51, 149.10, 130.47, 127.34, 120.28, 115.20, 114.64, 102.67, 55.35. HRMS (ESI-TOF) m/z [M+H]$^+$: calcd for $C_{13}H_{12}NO_3^+$ 230,0812, found 230.0822.

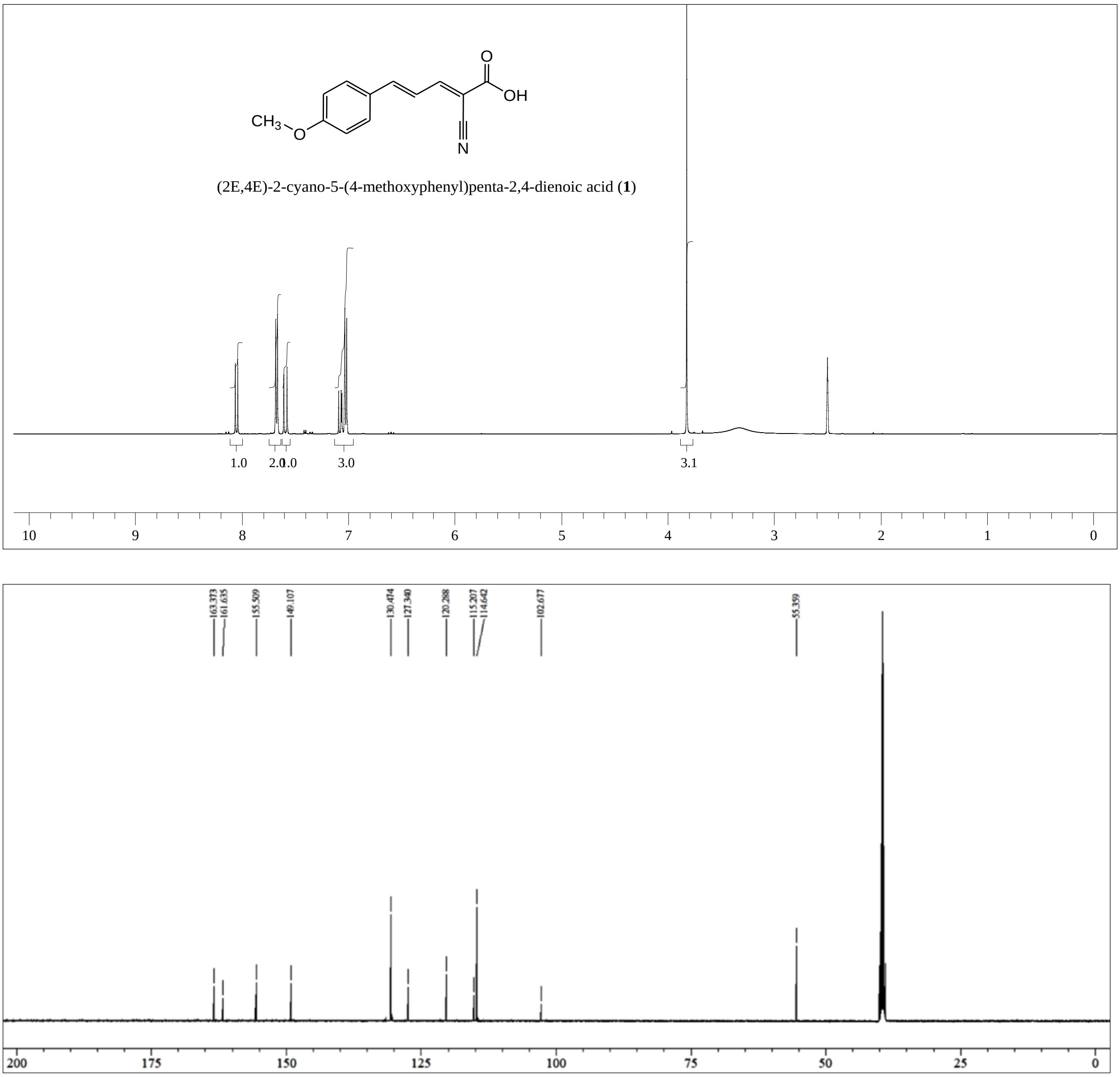

Figure S3. ^{1}H-NMR and ^{13}C-NMR spectra of the compound 1.

(2E,4E)-2-cyano-5-(4-nitrophenyl)penta-2,4-dienoic acid (**2**). This acid was obtained as a bright yellow solid in a 72% of yield. ^{1}H-NMR (400 MHz, DMSO-D6), δ: 7.37 (dd, 1 H, J= 15.65 and 11.74 Hz, vinyl proton), 7.73 (d, 1 H, J= 15.65 vinyl proton), 7.97 (d, 2 H, J= 8.80 Hz, aromatic protons), 8.10 (d, 1 H, J= 11.74 vinyl proton), 8.27 (d, 2 H, J= 8.80 Hz, aromatic protons); ^{13}C-NMR (DMSO-D6) δ: 162.73, 153.75, 147.93, 145.47, 140.85, 129.37, 126.51, 124.05, 114.64, 107.38. HRMS (ESI-TOF) m/z [M+H]$^{+}$: calcd for $C_{12}H_{9}N_{2}O_{4}^{+}$ 245,0557, found 245,0568.

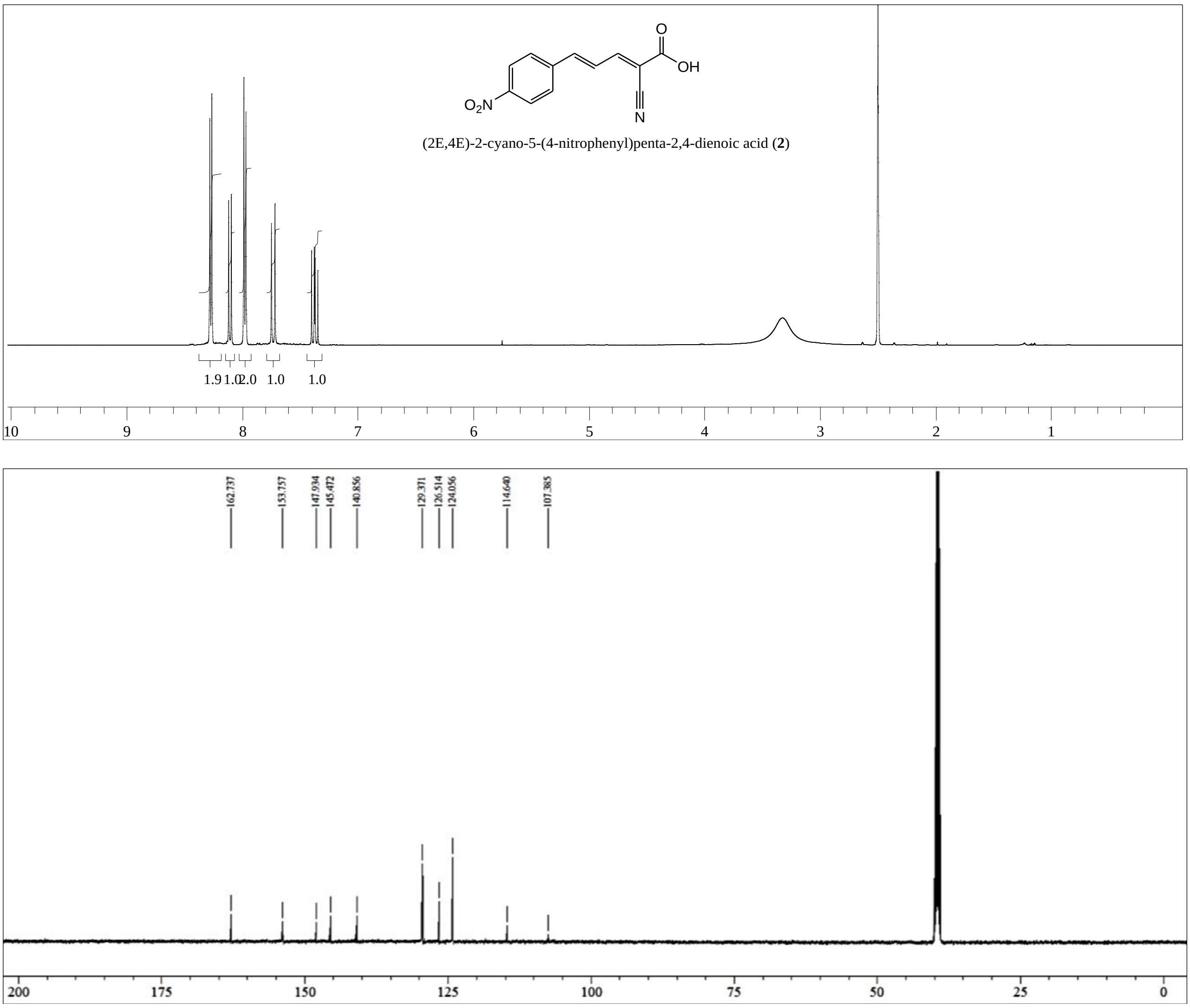

Figure S4. ^{1}H-NMR and ^{13}C-NMR spectra of the compound 2.

*(2E,4E)-2-cyano-5-(4-(dimethylamino)phenyl)penta-2,4-dienoic acid (**3**)*. This acid was obtained as a dark solid (mp 221–223 °C) in a 55% of yield. ^{1}H-NMR (DMSO-D6), δ: 3.02 (s, 6 H, methyl groups), 6.75 (d, 2 H, J= 8.94 Hz, aromatic protons), 6.91 (dd, 1 H, J= 14.90 and 11.92, vinyl proton), 7.51 (d, 1 H, J= 14.90, vinyl proton), 7.53 (d, 2 H, H= 8.94, aromatic protons), 7.99 (d, 1 H, J= 11.92 Hz); Lit. [1].

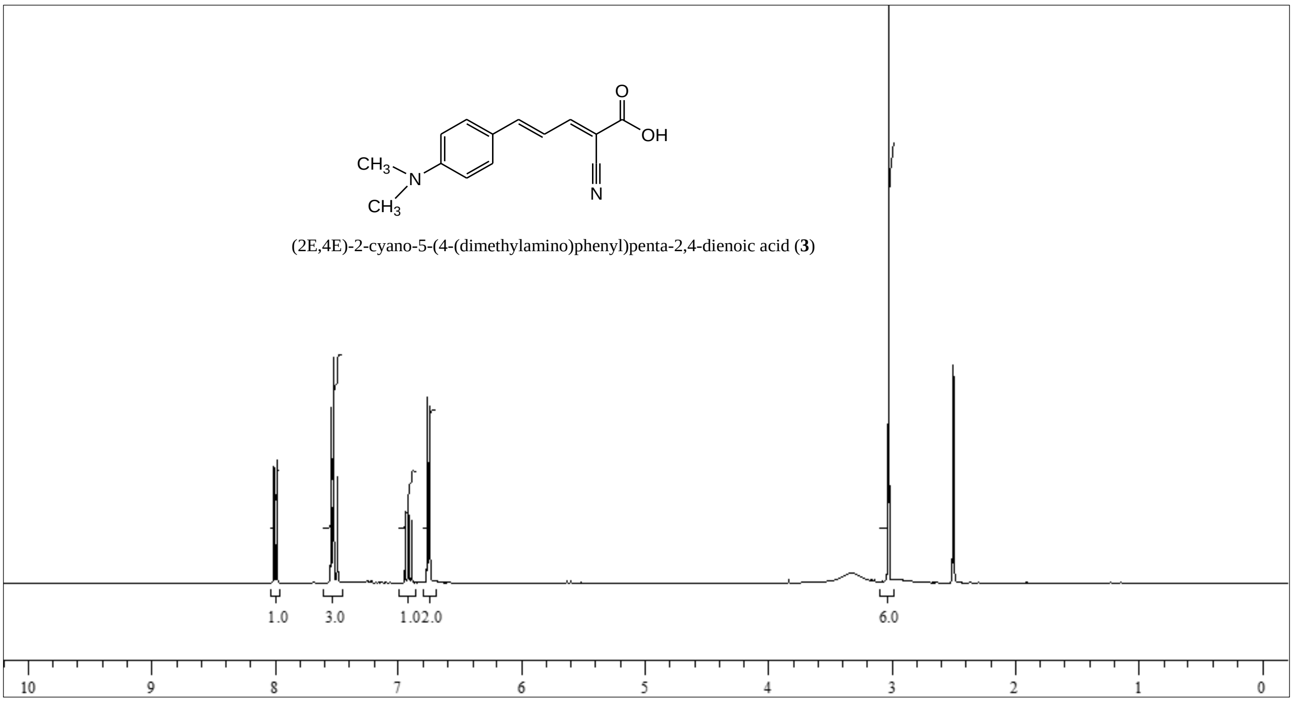

Figure S5. ^{1}H-NMR spectrum of the compound 3.

[1] K. Hara, M. Kurashige, S. Ito, A. Shinpo, S. Suga, K. Sayama, H. Arakawa *Chem. Commun.*, 2003, 252–253.

*(2E,4E)-2-cyano-5-phenylpenta-2,4-dienoic acid (**4**)*. ^{1}H-NMR (DMSO-D6), δ: 7.20 (dd, 1 H, J= 15.30 and 11.52 Hz, vinyl proton), 7.45 (m, 3 H, aromatic protons), 7.63 (d, 1 H, J= 15.30, vinyl proton), 7.70 (m, 2 H, aromatic protons), 8.07 (d, 1 H, J= 11.52 Hz, vinyl proton). Lit. [2].

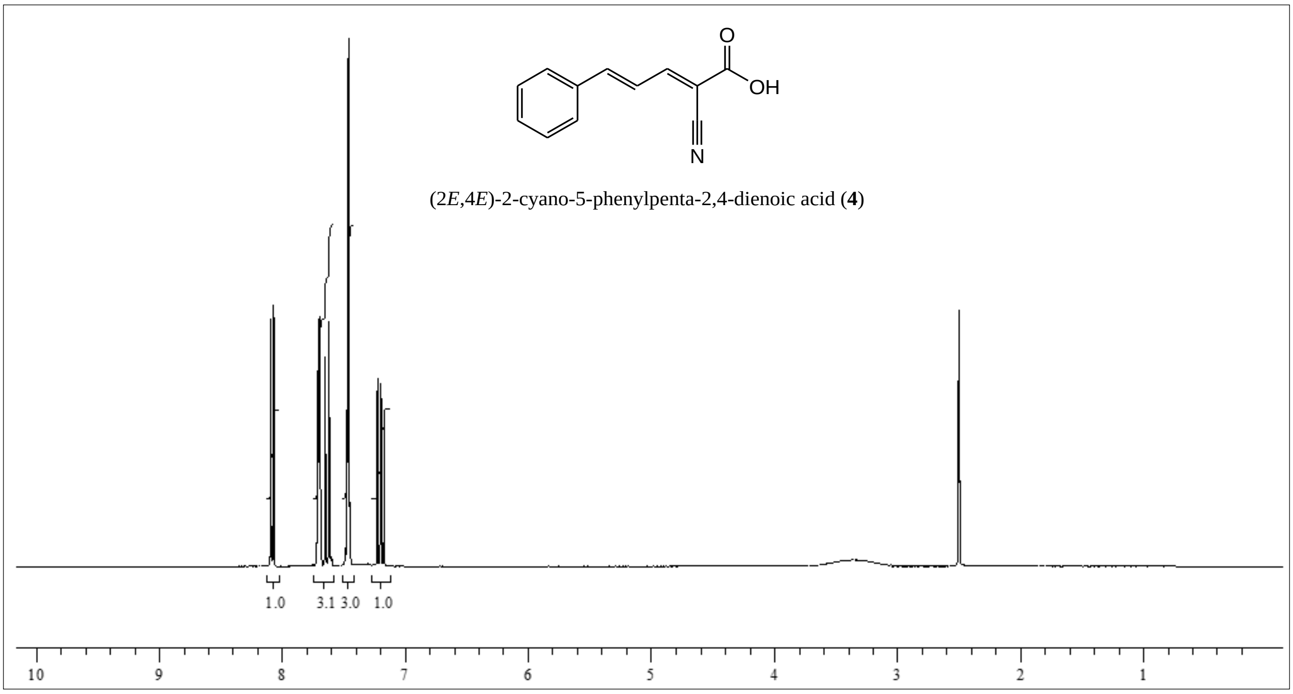

Figure S6. ^{1}H-NMR spectrum of the compound 4.

[2] A. Monopoli, A. Nacci, T. Cataldi, C. D. Calvano *Molecules*, 2020, 25 (24), 6054.

*(2E,4E)-5-(4-chlorophenyl)-2-cyanopenta-2,4-dienoic acid (**5**)*. This acid was obtained as a deep yellow solid (m.p. 235-236°C) in a 68% of yield. ^{1}H-NMR (400 MHz, DMSO-D6), δ: 7.21 (dd, 1 H, J= 15.30 and 11.72 Hz, vinyl proton), 7.51 (d, 2 H, J= 8.54 Hz, aromatic protons), 7.62 (d, 1 H, J= 15.30 Hz, vinyl proton), 7.73 (d, 2 H, J= 8.54 Hz, aromatic protons), 8.07 (d, 1 H, J= 11.52 Hz, vinyl proton); ^{13}C-NMR (DMSO-D6) δ: 163.02, 154.61, 147.18, 135.30, 133.55, 130.07, 129.10, 123.35, 114.85, 105.26. HRMS (ESI-TOF) m/z $[M+H]^+$: calcd for $C_{12}H_9ClNO_2^+$ 234,0316, found 234,0328.

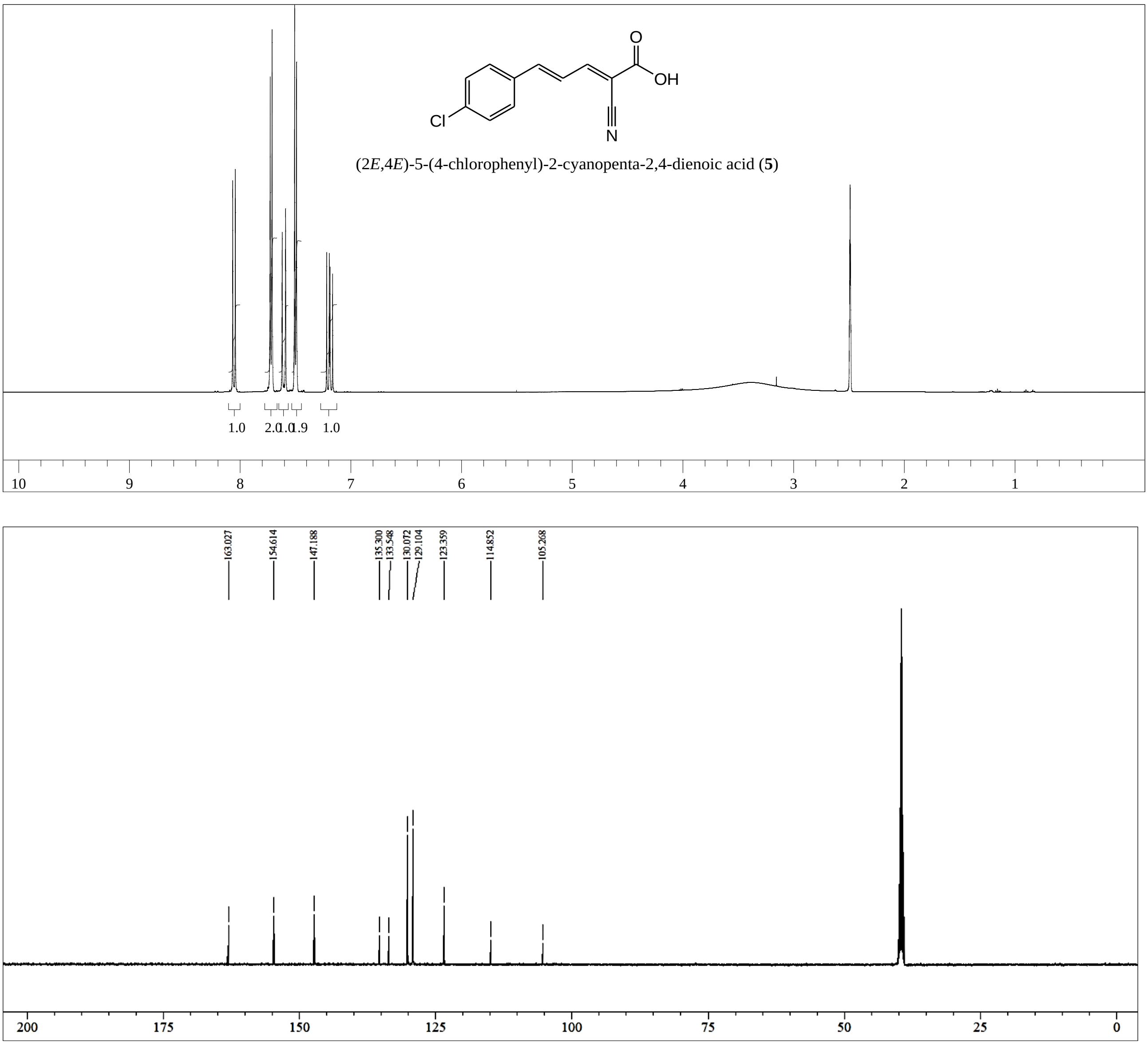

Figure S7. ^{1}H-NMR and ^{13}C-NMR spectra of the compound 5.

(E)-2-cyano-3-(6-methoxynaphthalen-2-yl)acrylic acid (**6**). This acid was obtained as a yellow solid (m.p. 248-250°C) in a 75% of yield. ^{1}H-NMR (400 MHz, DMSO-D6), δ: 3.93 (s, 3 H, methoxy), 7.28 (dd, 1 H, J= 8.94 and 2.38 Hz), 7.43 (d, 1 H, J= 2.38 Hz), 7.94 (d, 1 H, J= 9.14 Hz), 7.97 (d, 1 H, J= 8.74 Hz), 8.18 (dd, 1 H, J= 8.74 and 1.59 Hz), 8.41 (s, 1 H), 8.48 (s, 1 H); ^{13}C-NMR δ: 163.45, 159.73, 154.15, 136.59, 133.94, 130.88, 127.61, 127.60, 126.71, 125.12, 119.82, 116.46, 106.26, 101.89, 55.42. HRMS (ESI-TOF) m/z $[M+H]^+$: calcd for $C_{15}H_{12}NO_3^+$ 254,0812, found 254,0824.

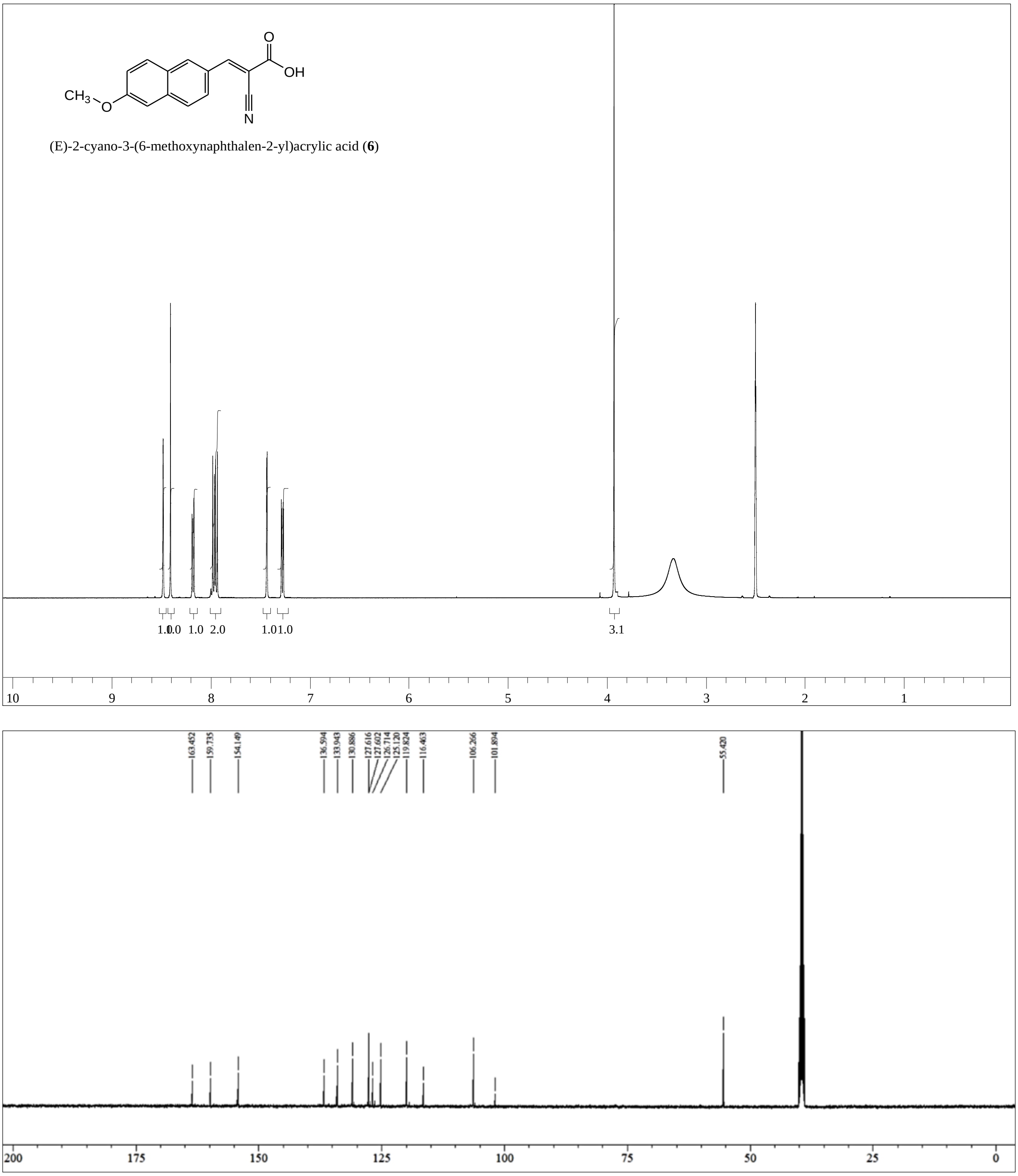

Figure S8. ^{1}H-NMR and ^{13}C-NMR spectra of the compound 6.

(E)-2-cyano-3-(naphthalen-2-yl)acrylic acid (7). This acid was obtained as a white solid in a 86% of yield. ^{1}H-NMR (DMSO-D6), δ: 7.67 (m, 2 H), 8.02 (m, 2 H), 8.09 (d, 1 H, J= 8.80 Hz), 8.19 (dd, 1 H, J= 8.31 and 1.47 Hz), 8.48 (s, 1 H), 8.56 (s, 1 H). Lit. [3].

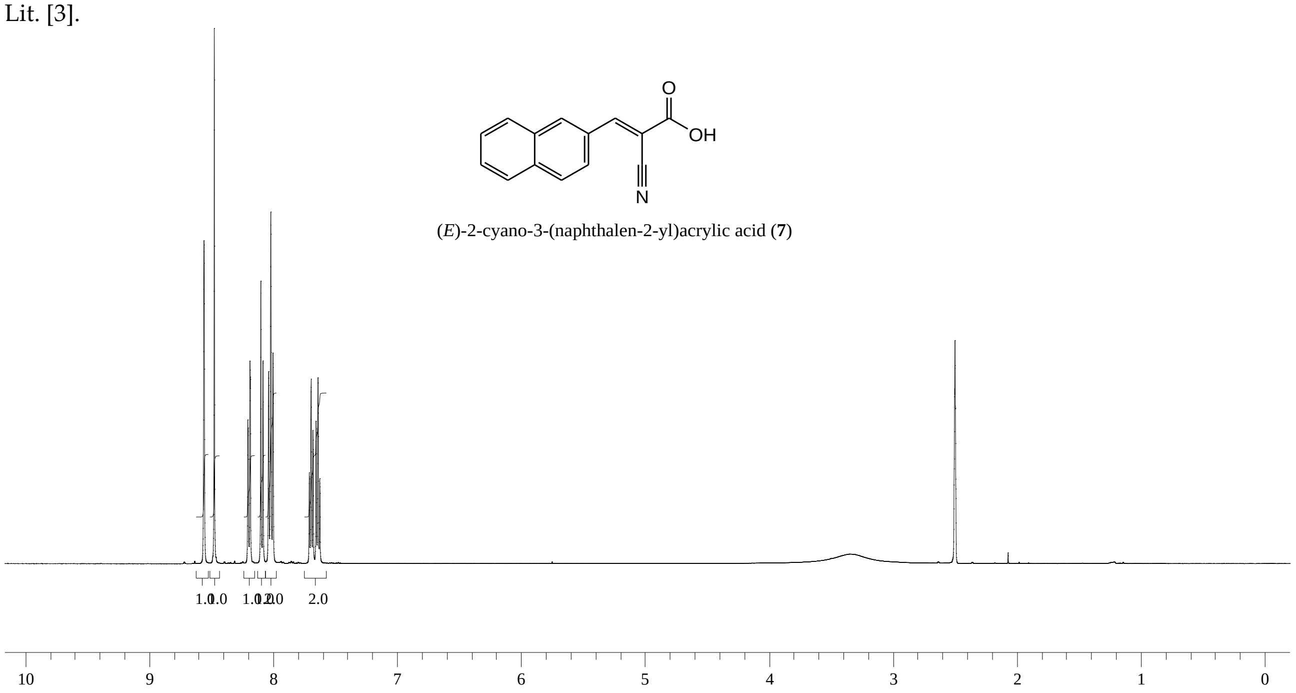

Figure S9. ^{1}H-NMR spectrum of the compound 7.

---

[3] T. Porta, C. Grivet, R. Knochenmuss, E. Varesio, G. Hopfgartner _J. Mass. Spectrom._ 2011, 46, 144–152

(2E,2′E)-3,3′-(1,4-phenylene)bis(2-cyanoacrylic acid) (8). This acid was obtained as a deep yellow in a 81% of yield. In this case, terephtaldehyde was condensed with cyanoacetic acid.

^{1}H-NMR (DMSO-D6), δ: 8.16 (s, 4 H), 8.39 (s, 2 H). ^{13}C-NMR (DMSO-D6) δ: 162.73, 152.54, 134.96, 130.77, 115.69, 106.23. HRMS (ESI-TOF) m/z $[M+H]^+$: calcd for $C_{14}H_9N_2O_4^+$ 269,0557, found 269,0569.

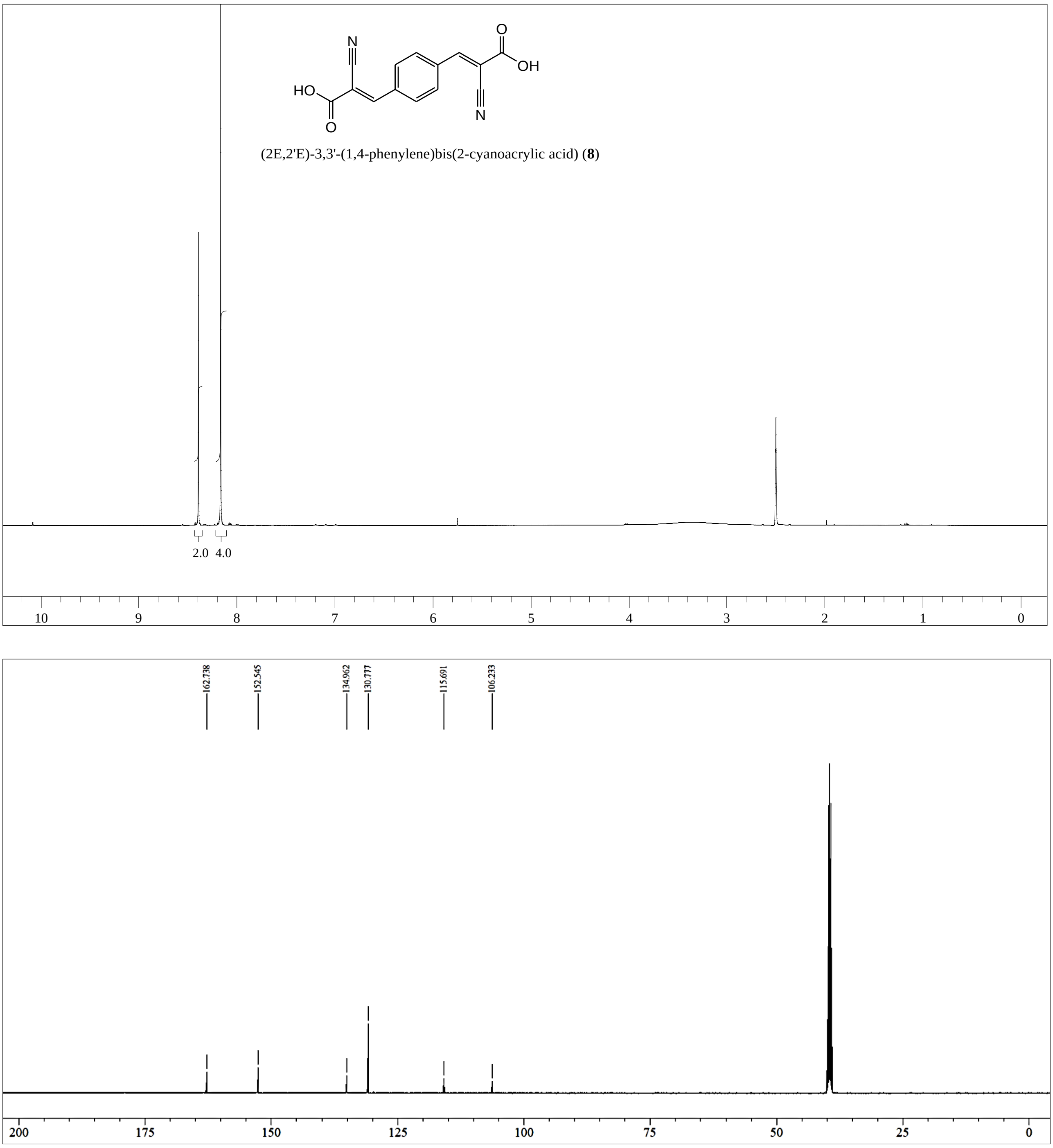

Figure S10. ^{1}H-NMR and ^{13}C-NMR spectra of the compound 8.

*(E)-3-(4′′-((E)-2-carboxy-2-cyanovinyl)-[1,1′:4′,1′′-terphenyl]-4-yl)-2-cyanoacrylic acid (**9**)*. This acid was obtained as an orange solid (m.p. 264-265°C) in a 54% of yield. The matrix was obtained by a Knoevenagel condensation between cyanoacetic acid and [1,1′ :4′ ,1″ -terphenyl]-4,4″ -dicarbaldehyde. This dicarbaldehyde was synthetized starting from 1,4-dibromo benzene and (4-formylphenyl)boronic acid following a literature procedure (*Synthetic Communications*, **2005**, vol. 35, 2, p. 161 - 167)

^{1}H-NMR (DMSO-D6), δ: 7.48 (m, 4 H, AA′BB′ spin system), 7.83 (d, 4 H, J= 8.31 Hz), 8.08 (d, 4 H, J= 8.31 Hz), 8.31 (s, 2 H, vinyl protons). ^{13}C-NMR (DMSO-D6) δ: 163.28, 153.27, 141.64, 136.98, 131.18, 130.53, 127.70, 127.03, 116.34, 102.83.

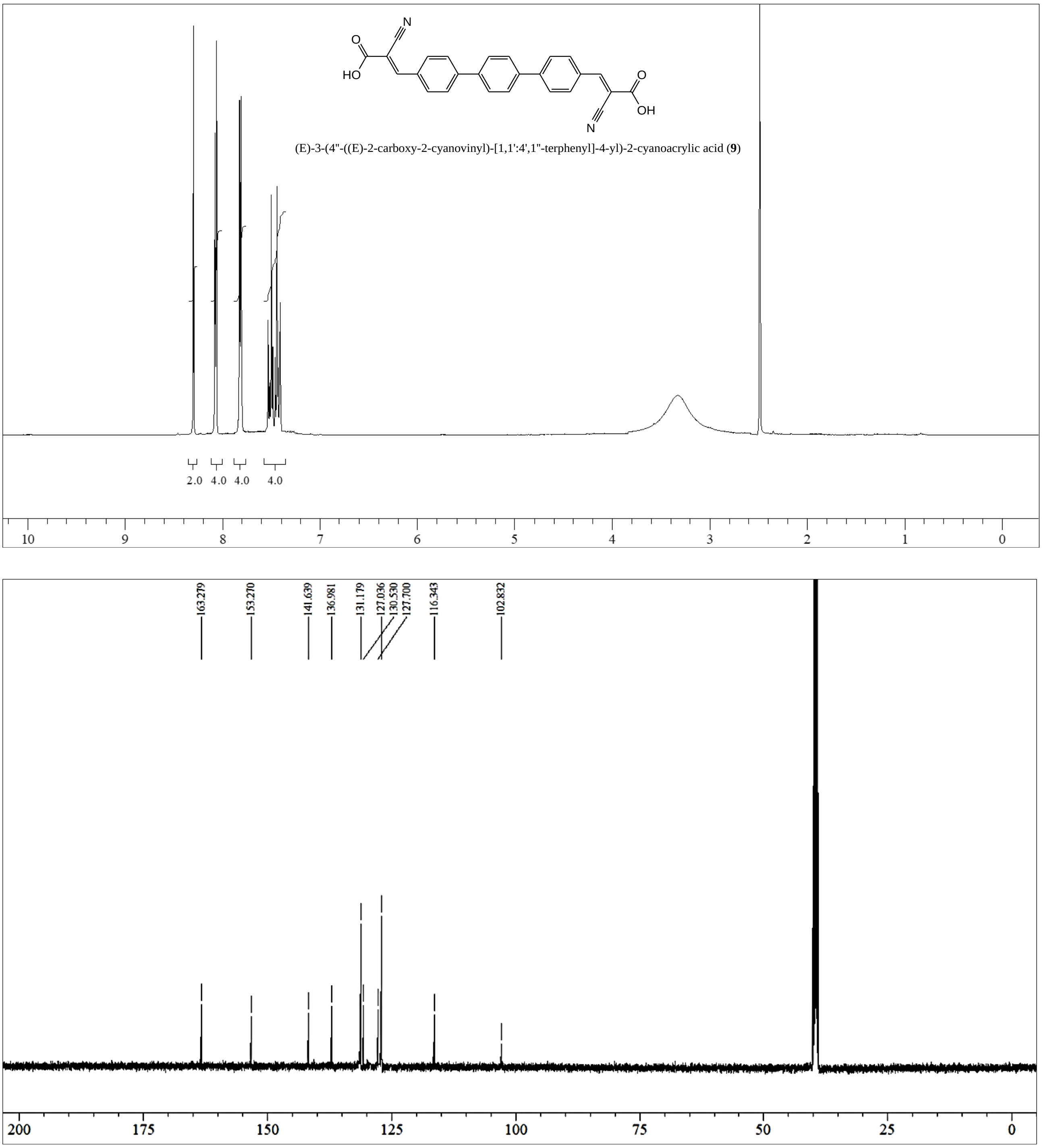

Figure S11. ^{1}H-NMR and ^{13}C-NMR spectra of the compound 9.

*(E)-2-cyano-3-(quinolin-4-yl)acrylic acid (**10**)*. This acid was obtained as a brown solid in a 65% of yield. ^{1}H-NMR (400 MHz, DMSO-D6), δ: 7.73 (t, 1 H, J= 7.33), 7.88 (m, 2 H,), 8.05 (d, 1 H, J= 7.83 Hz), 8.14 (d, 1 H, J= 8.31 Hz), 8.99 (s, 1 H), 9.08 (d, 1 H, J= 3.91 Hz); ^{13}C-NMR (DMSO-D6) δ: 161.98, 150.99, 150.22, 147.64, 137.46, 130.24, 129.62, 127.78, 124.57, 124.10, 120.17, 114.71, 112.17. HRMS (ESI-TOF) m/z [M+H]$^{+}$: calcd for $C_{13}H_9N_2O_2^+$ 225,0659, found 225,0648.

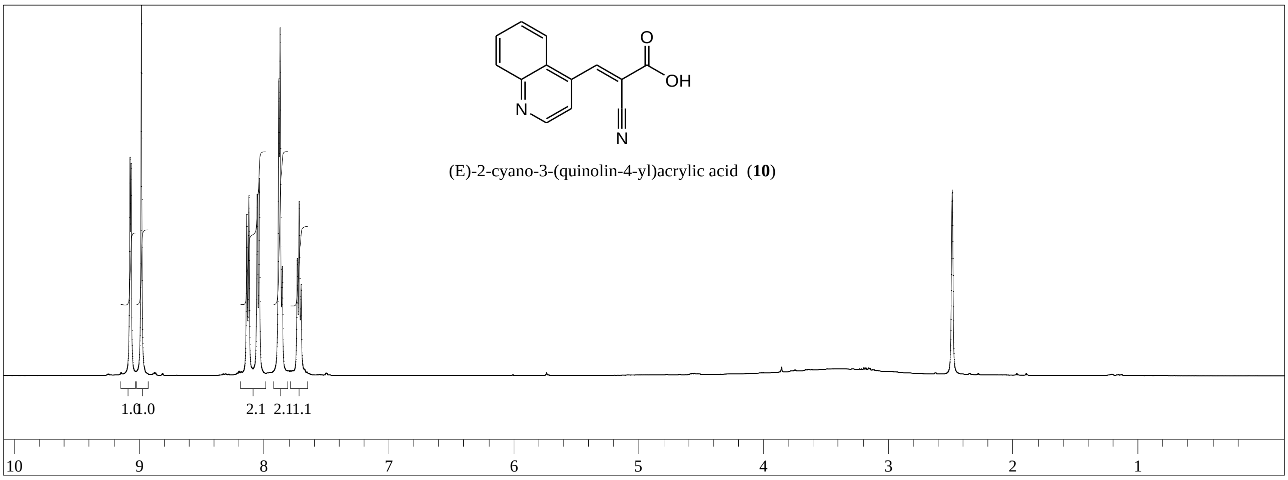

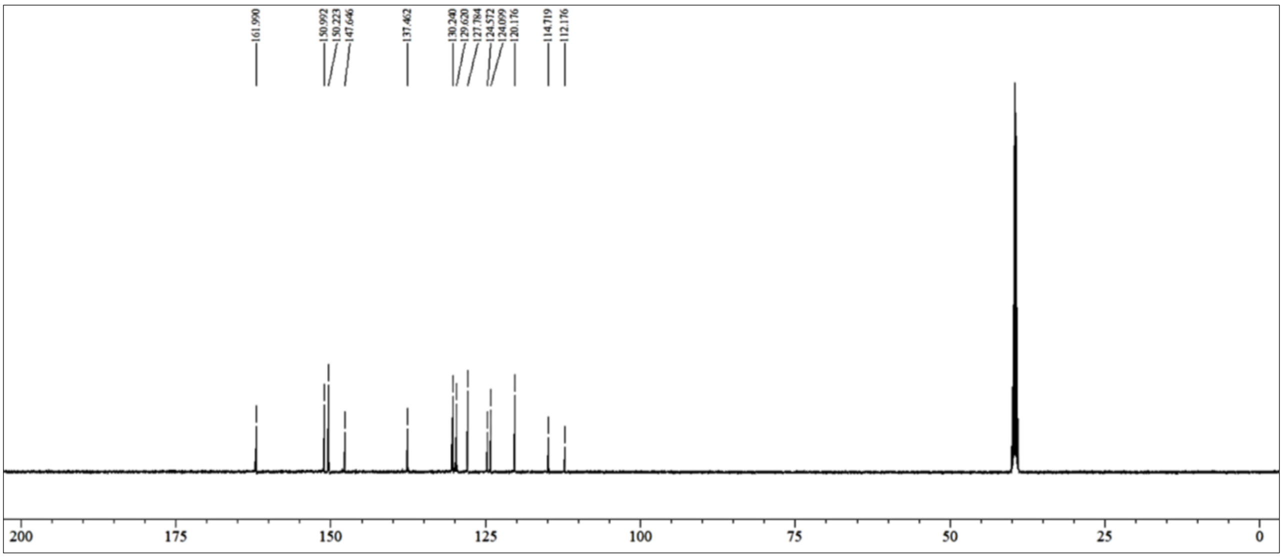

Figure S12. ^{1}H-NMR and ^{13}C-NMR spectra of the compound 10
